# Characterizing RecA-Independent Induction of Shiga toxin2-Encoding Phages by EDTA Treatment

**DOI:** 10.1371/journal.pone.0032393

**Published:** 2012-02-29

**Authors:** Lejla Imamovic, Maite Muniesa

**Affiliations:** Department of Microbiology, University of Barcelona, Barcelona, Spain; The University of Hong Kong, Hong Kong

## Abstract

**Background:**

The bacteriophage life cycle has an important role in Shiga toxin (Stx) expression. The induction of Shiga toxin-encoding phages (Stx phages) increases toxin production as a result of replication of the phage genome, and phage lysis of the host cell also provides a means of Stx toxin to exit the cell. Previous studies suggested that prophage induction might also occur in the absence of SOS response, independently of RecA.

**Methodology/Principal Findings:**

The influence of EDTA on RecA-independent Stx2 phage induction was assessed, in laboratory lysogens and in EHEC strains carrying Stx2 phages in their genome, by Real-Time PCR. RecA-independent mechanisms described for phage λ induction (RcsA and DsrA) were not involved in Stx2 phage induction. In addition, mutations in the pathway for the stress response of the bacterial envelope to EDTA did not contribute to Stx2 phage induction. The effect of EDTA on Stx phage induction is due to its chelating properties, which was also confirmed by the use of citrate, another chelating agent. Our results indicate that EDTA affects Stx2 phage induction by disruption of the bacterial outer membrane due to chelation of Mg^2+^. In all the conditions evaluated, the pH value had a decisive role in Stx2 phage induction.

**Conclusions/Significance:**

Chelating agents, such as EDTA and citrate, induce Stx phages, which raises concerns due to their frequent use in food and pharmaceutical products. This study contributes to our understanding of the phenomenon of induction and release of Stx phages as an important factor in the pathogenicity of Shiga toxin-producing *Escherichia coli* (STEC) and in the emergence of new pathogenic strains.

## Introduction

Shiga toxin-producing *Escherichia coli* (STEC) are important food- and water-borne pathogens that are responsible for gastrointestinal disease throughout the world [1]–[4]. In some cases STEC infection can lead to life-threatening complications, including neurological damage and hemolytic-uremic syndrome [3], [4]. Several hundred STEC serotypes have been isolated and some of them, like O157:H7, are highly virulent in humans [2], [3]. Although STEC possess a broad range of virulence factors, attaching and effacing (A/E) lesions and the ability to produce one or more toxins of the Shiga toxin (Stx) family are the hallmarks of infections caused by STEC [2]–[5]. Stxs are AB_5_ holotoxins that catalyze the removal of a specific adenine residue from the 28 S rRNA of the 60 S ribosomal subunit, resulting in inhibition of protein synthesis in the target cell [4], [5]. Two broad groups of Stxs have been reported: Stx1 and Stx2. While Stx1 show little sequence variation, Stx2 are a heterogeneous group and several variants have been described [5]. STEC strains producing Stx2 or Stx2c, alone or in combination, are more commonly associated with severe diseases than those producing only Stx1 [6], [7].

The genes encoding Stx are carried on the genome of lambdoid type phages (Stx phages) [1], [8]–[11]. These phages are highly diverse mobile genetic elements that transfer the *stx* gene not only to commensal and pathogenic *E. coli* but also to other members of the *Enterobacteriaceae* family [12]–[16]. Stx genes are located in the late gene region downstream of the late promoters and upstream of the phage lysis cassette [17]. In a lysogen, the expression of the *stx* genes is controlled by the phage repressor [10], [18], [19]. Nevertheless, a small fraction of lysogens is induced even in the absence of any inducing agent, a process known as spontaneous phage induction [20]. Stx phages are spontaneously induced more readily than other lambdoid phages that do not carry *stx* genes [20], [21]. Activation of the SOS response due to conditions that cause DNA damage or inhibition of DNA replication triggers significantly higher levels of Stx phage induction [22]. Exposure to SOS-inducing agents, which include several classes of antibiotics, UV light, high hydrostatic pressure and H_2_O_2_, dramatically increases toxin and phage induction [23]–[27]. This occurs via the well-characterized molecular mechanism of RecA-mediated cleavage of phage repressor [23], [28], [29].

However, RecA-independent induction also occurs in phages infecting *E. coli* [30]–[32]. In phage λ, induction does not always imply repressor cleavage by RecA. In this case, the mechanism involves RcsA, a regulator of colanic acid synthesis, and DsrA [30], [32], a small regulatory RNA that, among other functions, prevents the degradation of RcsA [30], [33]. The observation by Muniesa et al. [8], [16] that free Stx2 phages are present in the culture of *E. coli recA*-negative strain DH5α raised questions regarding the signals and mechanisms that trigger Stx phage induction in the absence of RecA. Even though both dissemination of *stx* genes and regulation of their expression are of strong interest for public health, the signals or mechanisms that induce Stx2 phages, independently of RecA-promoted phage repressor cleavage, have not yet been elucidated.

EDTA is a common chelating agent and antioxidant that is added to foods, cosmetics and pharmaceutical products [34]. It is also used to treat acute hypercalcemia and lead poisoning [34], [35]. In addition, several authors have investigated the use of EDTA as an antimicrobial agent against pathogens such as *E. coli* O157:H7 [36], [37]. As an antimicrobial agent, EDTA acts by disrupting the structure of the outer membrane [38]. In doing so, EDTA activates three known envelope stress responses that are responsible for the monitoring and maintenance of cell envelope integrity in *E. coli*: RcsBC, CpxAR and BaeSR [39]. In this study, we investigated the effect of EDTA on RecA-independent Stx2 phage induction, as schematized in Figure S1. We assessed: i) the possible role of factors involved in RecA-independent induction of λ, RcsA and DsrA; ii) the role of the three envelope stress responses triggered by EDTA; iii) the effect of chelation; and iv) the role of pH in Stx2 phage induction.

## Methods

### Bacterial strains, bacteriophages and plasmids used in this study

The relevant characteristics of the strains and plasmids used in this study are listed in Table 1. *E. coli* O157:H7 strain EDL933 [1] is considered to be the reference serotype for enterohemorrhagic O157:H7 isolates. Phage 933 W [1], carrying the *stx*_2_ operon was originally isolated from strain EDL933. The characteristics of phage 933 W have been described previously [1], [10], [17], [19], [23]. Two laboratory strains of *E. coli* K12, a *recA*-positive strain C600 [23], and *recA*-negative strain DH5α (Gibco-BRL, Eggenstein, Germany), were used as a host for Stx2-encoding phage 933 W.

**Table 1:** Strains and plasmids used in this study.

Strain	Relevant characteristic	Reference
*E. coli* DH5α	Laboratory strain K12, *recA*-negative	Gibco-BRL
*E. coli* O157:H7 EDL933	*stx*_1_^+^, *stx*_2_^+^, clinical isolate	[1], [49]
*E. coli* O157:H7 R95	*stx*_1_^−^, *stx*_2_^+^, clinical isolate	This study
*E. coli* O157:H7 R262	*stx*_1_^−^, *stx*_2_^+^, clinical isolate	This study
*E. coli* O157:H7 R268	*stx*_1_^−^, *stx*_2_^+^, clinical isolate	This study
*E. coli* O157:H7 R283	*stx*_1_^−^, *stx*_2_^+^, clinical isolate	This study
*E. coli* O157:H7 R467	*stx*_1_^−^, *stx*_2_^+^, clinical isolate	This study
*E. coli* O174:H-, strain VTB280	*stx*_1_^−^, *stx*_2_^+^, clinical isolate	This study
*E. coli* O26:H- strain VTB57	*stx*_1_^−^, *stx*_2_^+^, clinical isolate	This study
*E. coli* O2:H29 strain VTB59	*stx*_1_^−^, *stx*_2_^+^, clinical isolate	This study
*E. coli* O166:H28 strain VTB273	*stx*_1_^−^, *stx*_2_^+^, clinical isolate	This study
*E. coli* DH5α pkD46	Strain with gene Red recombinase in vector pKD46	[44]
*E. coli* C600 (933 W)	Lysogen with phage 933 W	[23]
*E. coli* DH5α(933 W)	Lysogen with phage 933 W	This study
*E. coli* C600Δ*recA::tet*	Isogenic C600 *recA* ^−^ mutant	This study
*E. coli* C600(933 W)Δ*recA::tet*	*recA* ^−^ mutant, 933 W lysogen	This study
*E. coli* C600Δ*recA::tet* +pGEM::*recA*	*recA* ^−^ mutant complemented with *recA*	This study
*E. coli* C600(933 W)Δ*recA::tet* +pGEM::*recA*	*recA* ^−^ mutant complemented with *recA*, 933 W lysogen	This study
*E. coli* DH5αΔ*baeS*Δ*baeR::tet*	BaeSR-mutant	This study
*E. coli* DH5αΔ*rcsB*Δ*rcsC::tet*	RcsBC-mutant	This study
*E. coli* DH5αΔ*cpxA*Δ*cpxR::tet*	CpxAR-mutant	This study
*E.coli* DH5α Δ*rcsA*Δ*dsrA::tet*	*rcsA*- and *dsrA*-mutant	This study
*E. coli* DH5α(933 W)Δ*baeS*Δ*baeR::tet*	BaeSR-mutant, 933 W lysogen	This study
*E. coli* DH5α(933 W)Δ*rcsB*Δ*rcsC::tet*	RcsBC-mutant, 933 W lysogen	This study
*E. coli* DH5α(933 W)Δ*cpxA*Δ*cpxR::tet*	CpxAR-mutant, 933 W lysogen	This study
*E.coli* DH5α(933 W)Δ*rcsA*Δ*dsrA::tet*	*rcsA*- and *dsrA*-mutant, 933 W lysogen	This study
**Plasmid**		
pkD46	Red recombinase	AY048746
pACYC184	Tetracycline resistance gene *(tet)*	X06403
pBAD-TOPO	High copy number plasmid	Invitrogen
pBAD-TOPO::378*stx*	*stx*_2_ gene incorporated for Real Time PCR detection	[45]
pGEM-T-easy	High copy number plasmid	Promega
pGEM-T-easy::*recA*	*recA* gene incorporated	This study

### Media and growth conditions

Cells were grown in LB (Luria-Bertani) broth or LB agar. AB minimal media were used for some analyses [40]. SOB (Super Optimal Define broth) and SOC (Salt-Optimized Carbon broth) media were used to prepare electrocompetent cells and for recovery after transformation, as described below. When necessary, antibiotics were added to the media at the following concentrations: tetracycline 10 µg ml^−1^ and ampicillin 100 µg ml^−1^.

Overnight cultures were diluted 1∶100 in LB broth and incubated under agitation (180 rpm) at 37°C to an OD_600_ of 0.3. Bacteria were collected by centrifugation at 3.000× *g* for 10 min. To evaluate the Stx2 phage induction bacteria were incubated in LB or LB with the addition of ethylenediaminetetraacetate (EDTA) (5 and 20 mM), mitomycin C (0.5 µg ml^−1^), 2′2 dipyridyl (2 mg ml^−1^), sodium citrate (0.2 M), ethylene glycol bis (β-aminoethyl ether)-*N*,*N*,*N*′,*N*′-tetraacetic acid (EGTA) (20 mM), 1,2-bis(*o*-aminophenoxy)ethane-*N*,*N*,*N*′,*N*′-tetraacetic acid (BAPTA) (4 mg ml^−1^), or N,N,N′,N′-tetrakis (2 pyridylmethyl) ethylenediamine (TPEN) (1 mg ml^−1^). Bacterial growth and the number of Stx2 phage inductions in the cultures were determined after 2, 4 or 6 h. To study the influence of pH on 933 W phage induction, the cultures were incubated for 4 h in LB broth that was adjusted at the corresponding pH (3.0, 4.0, 5.5, 7.0, 8.5 and 10.0). The effect of pH on Stx2 phage induction was evaluated in the same manner when mitomycin C or EDTA were used.

To determine whether the addition of Mg^2+^, Ca^2+^ or Fe^3+^ inhibited the effect of EDTA on phage induction, bacteria were incubated in LB with EDTA supplemented with 20 mM of MgSO_4_, CaCl_2_ or FeCl_3_. To evaluate whether the lack of Mg^2+^, Ca^2+^ or Fe^3+^ influenced Stx2 phage induction, DH5α(933 W) lysogen was adapted to growth in AB minimal media. After reaching an optical density OD_600_ of 0.3 bacterial cells were collected by centrifugation at 3000× *g* for 10 min. The cells were then incubated in AB medium with EDTA or AB medium lacking Mg^2+^, Ca^2+^ or Fe^3+^. Viable cell counts were determined by plating the appropriate dilution on LB agar. Induced Stx2 phages were quantified after phage DNA extraction using Real-Time PCR, as described below.

### Bacterial growth and preparation of phage lysates

Bacterial density was evaluated by measuring the OD_600_, plating the appropriate dilution onto LB agar and incubating overnight at 37°C. Phage lysates were prepared by filtration of bacterial cultures through low protein-binding 0.22-µl pore size membrane filters (Millex-GP; Millipore). Phage lysates were used to obtain phage DNA for the quantification of free Stx2 phages using Real-Time PCR, as described below.

### Phage DNA extraction

Five ml of the phage lysates was concentrated using Amicon tubes (Millipore) and then treated with DNase (100 Units ml^−1^) (Roche Diagnostics, Barcelona, Spain). This eliminated DNA that was not encapsidated in the phage particle. An aliquot of the phage lysate at this stage was used as a control in Real-Time PCR to confirm that bacterial or non-encapsidated DNA containing the *stx*_2_ had been removed from the sample. Phage DNA was isolated by proteinase K digestion and by phenol/chloroform (1∶1) (*v∶v*) treatment. The phenol/chloroform/phage lysate mixture was added to Phase Lock Gel tubes (5- Prime, VWR International, Madrid, Spain) and processed according to the manufacturer's instructions. DNA was precipitated using absolute ethanol and 3 M sodium acetate and collected by centrifugation. Phage DNA was eluted in 30 µl of water and evaluated by agarose gel (0.8%) electrophoresis. The bands were then visualized by ethidium bromide staining. The concentration and purity of the phage DNA extracted were determined using a NanoDrop ND-1000 spectrophotometer (NanoDrop Technologies, Thermoscientifics. Wilmington, USA).

### DNA techniques

Restriction digestion, plasmid purification, DNA cloning, ligation, classical PCR amplification and gel electrophoresis were carried out according to standard protocols [41] and manufacturers' instructions.

### Real-Time PCR

Standard curves were generated using pBAD-TOPO, a high copy number plasmid conferring ampicillin resistance (Invitrogen Corporation, Barcelona, Spain). pBAD-TOPO::378*stx* was constructed as follows: a fragment of *stx*_2_ gene was amplified by PCR using the primers UP378 and LP378. The resulting fragment was purified using a Qiagen Gel Purification Kit (Qiagen) and cloned into pBAD-TOPO according to the manufacturer's instructions. The vector containing the insert was purified from positive colonies using a Qiagen Plasmid Purification Kit (Qiagen Inc., Valencia, CA, USA). The insertion of the *stx*_2_ fragment was confirmed by PCR and sequencing using primers UP378/LP378 and pBADf/pBADr (Invitrogen S.A., Barcelona, Spain). The construct was quantified using a NanoDrop ND-1000 spectrophotometer. The reaction product was linearized by digestion with restriction endonuclease *Eco*RV (Promega Co., Madison, WI, USA), purified and then quantified again. The number of gene copies was calculated using the following formula: n°molecules µl^−1^ = (concentration of the construct×6.023×10^23^ molecules mol^−1^)/molecular weight. A Custom TaqMan (Applied Biosystems, Barcelona, Spain) set of primers and probe were designed to amplify the *stx*_2_ gene (Table 2) using the One-Step RT PCR System (Applied Biosystems, Barcelona, Spain). The *stx*_2_ gene was amplified in a 20-µl reaction mixture using the PCR Master Mix (Applied Biosystems, Barcelona, Spain). The reaction contained 9 µl of the phage DNA sample or quantified plasmid. Thermal cycler conditions were: an initial step of 10 min at 95°C followed by 40 cycles of 15 s of denaturing at 95°C and 60 s of annealing/extending at 60°C.

**Table 2:** Oligonucleotides used in this study.

Name	Sequence (5′- 3′)	Characteristics	Reference
Stx1-UP	CAGTTAATGTGGTGGCGAAGG	gene *stx*_1_-A+B	[16]
Stx1-LP	ACTGCTAATAGTTCTGCGCATC		
S2Aup	ATGAAGTGTATATTATTTA	*stx*_2_ *A* fragment	[9]
S2Alp	TTCTTCATGCTTAACTCCT		
GK3	TCAGCCCCATACGATATAAG	*stx*_2_ *B* fragment	[16]
GK4	TGGAGTGGTGAATCCGTTAG		
UP378	GCGTTTTGACCATCTTCGT	378 bp *stx*_2_ *A* fragment	[42]
LP378	ACAGGAGCAGTTTCAGACAG		
Tc-5	TCAGCCCCATACGATATAAG	gene *tet* (Tc)	[44]
Tc-3	TGGAGTGGTGAATCCGTTAG		
Tc-int	TGTCGGAATGGACGATAT	binding 5′of *tet* cassette	[44]
RR46 LP	GAGCTCTAAGGAGGTTAT	Red recombinase in pKD46	[44]
RR46-UP	GTGCAGTACTCATTCGTT		
pBADf	ATGCCATAGCATTTTTATCC	Confirmation of pBAD construct	Invitrogen
pBADr	GATTTAATCTGTATCAGG		
pGEM7up	TGTAATACGACTCACTAT	Confirmation of pGEM construct	Promega
RecX up	TCAGTCGGCAAAATTTCGC	*recA* gene	This study
RecAv lp	AGGTGCACTGAAAGCGGCTCG		
Tc-5RecX lp	CTAACGGATTCACCACTCCA CGCAGTAGCCTGACGACGCA	fragment of the *tet* gene for generation of Δ*recA* mutant	This study
Tc-3RecAvup	CTTATATCGTATGGGGCTGA GGACCTGCGCGTATTCGCCA		
Tc-5RcsB	CTTATATCGTATGGGGCTGAGCGAATACCGAACAAGACTA	Fragment of *tet* gene for Δ*rcsB*Δ*rcsC* mutant	This study
Tc-3RcsC	CTAACGGATTCACCACTCCA TCACTACCGCTCTGCCCTGC		
RcsAup	ATGTCAACGATTATTATGGAT	fragment between *rscA* and *dsrA*	This study
YedPlp	TGGCTAATACCTGAGATGAT		
Tc-5RcsA	CTTATATCGTATGGGGCTGA CGCTGACTGTTAGAAGCATC	fragment of *tet* gene for Δ*rcsA*Δ*dsrA* mutant	This study
Tc-3YedP	CTAACGGATTCACCACTCCA ATGTTTTCAATTCAACAACC		
CpxAup	GAATAACGCAGAGCATTAC	CpxAR phosphorelay system	This study
CpxPlp	GAACTGACTGCCAGCGTTGA		
Tc-5CpxA	CTTATATCGTATGGGGCTGA AGTTGTGGAGTGAAGTGCTG	fragment of *tet* gene for Δ*cpxA*Δ*cpR* mutant	This study
Tc-3CpxP	CTAACGGATTCACCACTCCA TGAAGCCTTCCATCTCGAG		
BaeSup	TGAAGTTCTGGCGACCCGGTAT	BaeSR phosphorelay system	This study
BaeRlp	CTAAACGATGCGGCAGGCGTCG		
Tc-5BaeS	CTTATATCGTATGGGGCTGA CTGAAAGACAAAGCGATCAT	fragment of *tet* gene for Δ*baeS*Δ*baeR* mutant	This study
Tc-3BaeR	TAACGGATTCACCACTCCA GCGTAGTAACCGACCGCACC		
STX-Any f	ACGGACAGCAGTTATACCACTCT	Real-Time PCR for *stx*_2_ gene	[45]
STX-Any r	ACGTTCCGGAATGCAAATCAG		
STX-Any M	FAM- CCAGCGCTGCGACACG-NFQ		

### Construction of lysogens

Phage 933 W, induced from C600(933 W) [1], was used to generate the lysogens according to the protocol described by Muniesa et al. [8]. Briefly, C600(933 W) lysogen was grown in LB broth at 37°C to the exponential growth phase and induced with mitomycin C added to a final concentration of 0.5 µg ml^−1^. After overnight incubation, the phage lysate was prepared by filtration through low-protein-binding 0.22 µm membrane filters and treated with DNase (10 U ml^−1^) (Roche Diagnostics). One ml of the phage lysate was added to a host strain grown to the exponential phase. After an overnight incubation at 37°C serial dilutions were plated onto LB agar and the lysogens were detected by colony hybridization and confirmed by PCR.

### Preparation of digoxigenin-labeled Stx2-A gene probe

The amplified DNA fragment of the A subunit of the *stx*_2_ gene obtained with primers UP378/LP378 [42] was labeled with digoxigenin and used as a probe. This probe allows specific detection of the *stx*_2_ gene and has been used previously to detect Stx phages [8]. The probe was labeled by incorporating digoxigenin-11-uridine-triphosphate (Roche Diagnostics) during PCR as described previously [8].

### Colony blot

To screen for lysogens with 933 W phages, the colonies were transferred to a nylon membrane (Hybond-N+; Amersham Pharmacia Biotech, Spain) according to standard procedure [41]. Hybridization was performed as described elsewhere [8]. Briefly, membranes were treated with prehybridization solution at 68°C for 2 h and then hybridized at 65°C with digoxigenin-labeled *stxA*_2_ probe as described previously. Stringent hybridization was accomplished using the DIG-DNA Labeling and Detection Kit (Roche Diagnostics) according to the manufacturer's instructions.

### Construction of C600Δ*recA*::*tet*, DH5αΔ*baeS*Δ*baeR*::*tet*, DH5αΔ*rcsB*Δ*rcsC*::*tet*, DH5αΔ*cpxA*Δ*cpxR*::*tet* and DH5αΔ*rcsA*Δ*dsrA*::*tet* recombinant strains

The Red recombinase system was used to generate mutants, with deletion of the target genes by insertion of the *tet* cassette, following the protocol of Datsenko and Wanner [43] with some modifications [44]. The primers used to generate the mutants are listed in Table 2. Strain C600 was selected for generation of an isogenic mutant, constructed by replacement of a fragment containing *recA* (1,564 bp) by *tet* gene (1,180 bp), generating strain C600(933 W)Δ*recA*::*tet*. The *recA* mutation was complemented by cloning *recA* in a pGEM-T-easy cloning vector (Promega), and subsequent transformation of the construct in C600(933 W)Δ*recA*::*tet*. *recA*-negative strain DH5α was used to generate mutations in the three envelope stress pathways (BaeSR, RcsBC and CpxAR). The strains carrying a recombinant plasmid pkD46 were incubated in 50 ml SOB medium and the cells were collected by centrifugation at 3000× *g* for 10 min. After washing the cells several times in ice-cold water, they were suspended in 200 µl of water, then mixed with 20 µl of the purified PCR product obtained via a 3-step PCR protocol [44], transferred to a 0.2-cm electroporation cuvette (Bio-Rad) and electroporated (2.5 kV, 25 F, 200Ω). Following electroporation, the cells were incubated in 5 ml of SOC medium for 3 h at 37°C and plated onto LB agar supplemented with tetracycline. The presence of the inserted fragment was confirmed by PCR. The mutants obtained were lysogenized with the 933 W phage and used to evaluate the role of mutated genes in Stx2 phage induction.

### Evaluation of Stx production

To determine the increase in the production of Stx after EDTA treatment, a cytotoxicity assay was used. Stx activity of the supernatants of the lysogens C600(933 W), DH5α(933 W) and strain EDL933 with and without EDTA or mitomycin C was determined by using serial dilutions (1/2, 1/4, 1/8, 1/10, 1/20 and 1/50) of filtered supernatants that were incubated with 4×10^4^ Vero cells ml^−1^ at 37°C in a 5% CO_2_ atmosphere. Purified Stx2 from Toxin Technology, Inc. (Sarasota, FL.) served as a positive control. Cells were incubated for 72 h and evaluated every 12 h. The amount of toxin contained in the last dilution of the sample in which 50% or more of the Vero cells had detached from the plastic, as assessed by direct microscope observation and confirmed by A620, was considered to be the 50% cytotoxic dose (CD_50_).

### Electron microscopy

The Stx phages present in the phage lysates obtained after induction of each lysogen were used for electron microscopy studies. A drop of this phage suspension was deposited on copper grids with carbon-coated Formvar films and stained with 2% KOH phosphotungstic acid (pH 7.2) for 2.5 min. Samples were examined in a Hitachi E.M. 800 electron microscope operating at 80 kV.

### Statistical analysis

The Statistical Package for Social Science software (SPSS) was used for computation of the data and statistical tests. Evaluation of the differences between phage-inducing conditions was performed by one-way analysis of variance (ANOVA). Evaluations were based on a 5% significance level (*P* 0.05).

## Results

### Quantification of 933 W phage release

The Stx phages described to date carry one copy of the *stx* operon in their genome [11]. This allows easy determination of phage number from isolated phage DNA, since one *stx* gene copy (GC) corresponds to one Stx phage particle. In order to assess the number of phages released in the culture we used Real-Time PCR. This technique allows detection of phages in a selected medium even when they are present at very low levels. The limit of detection when this set of primers for Real-Time PCR (Table 2) was used for Stx2 phage detection was 5.29 GC [45]. Therefore, this technique allowed us to monitor even very small changes in the number of Stx2 phages released.

### EDTA as an inducing agent

**Figure 1:**
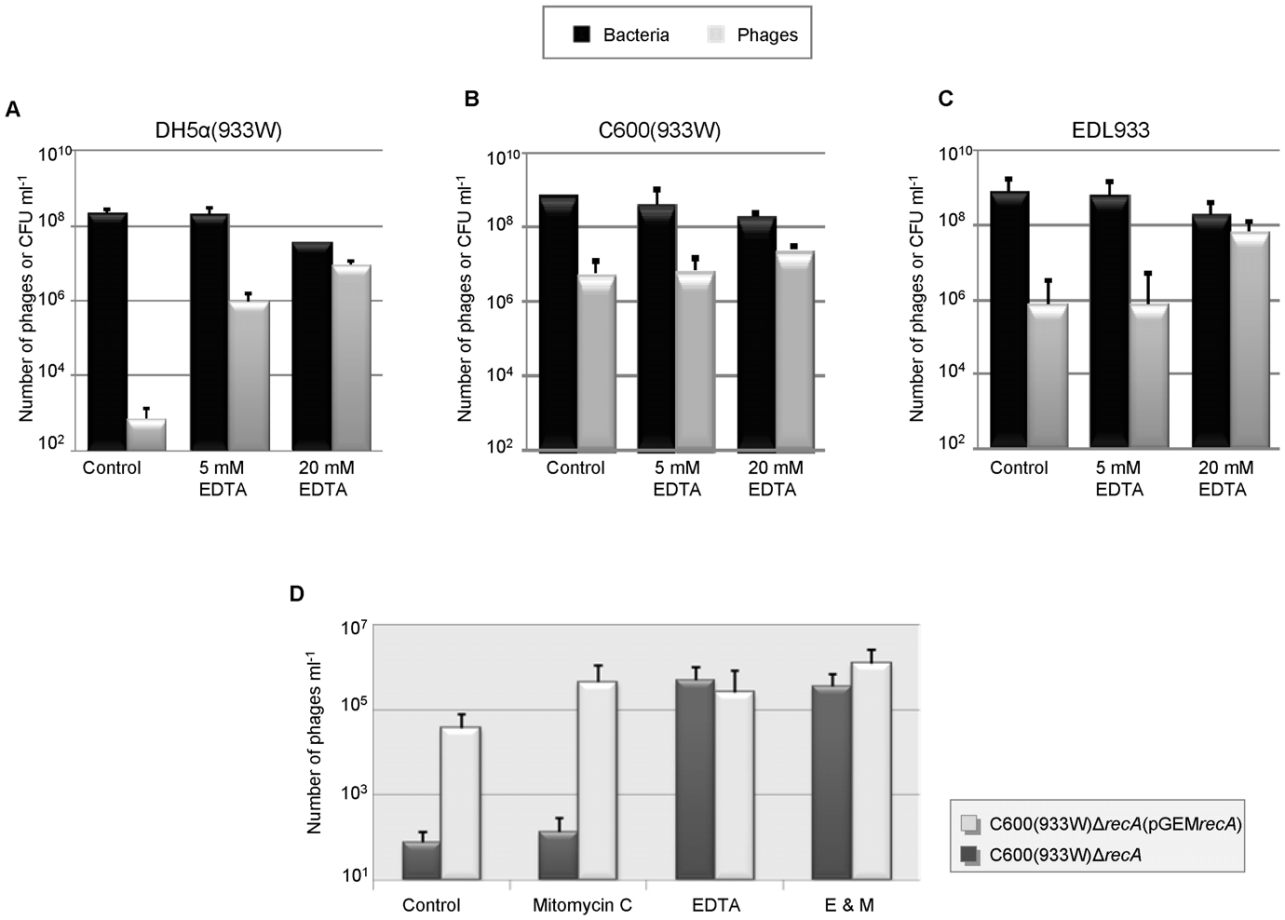
The dose response to EDTA on *recA*-independent induction. The effect of EDTA was evaluated in two laboratory *E. coli* lysogens of 933 W: DH5α(933 W) (A) and C600(933 W) (B), in *E. coli* O157:H7 EDL933 (C) in a 933 W lysogen *recA* isogenic mutant C600(933 W)Δ*recA*::*tet* alone and complemented with pGEM::*recA* (D). The effect of EDTA on 933 W in terms of phage induction and the number of viable cells (ml^−1^) was evaluated after 4 h of incubation at 37°C. Data presented are the mean of three experiments. Error bars indicate the standard deviation.

To establish the effects of EDTA on bacterial growth and phage induction and release, different concentrations of EDTA were tested (Figure 1A, 1B and 1C). The induction of Stx2 phages in control cultures was used as a background measure of spontaneous phage induction. Lysogens incubated with 5 mM of EDTA showed a significant increase in 933 W phages compared to the control cultures only in the DH5α(933 W) lysogen, with no significant effect on C600(933 W) or EDL933. The addition of 20 mM EDTA to the cultures reduced bacterial growth in all three lysogens. A difference in the number of Stx2 phages was also detected in cultures with a higher level of spontaneous phage induction. On the basis of these results, in order to observe the greatest effect we chose the concentration of 20 mM EDTA for further experiments.

The stability of the phage particles produced from the lysogens after EDTA treatment was assessed by electron microscopy, which showed intact phage particles with a short tail, corresponding to 933 W morphology [8]. The infectivity of the induced 933 W phages was confirmed after observing their ability to generate plaques of lysis on a monolayer of *E. coli* DH5α used as host strain.

Cytotoxicity assays performed with EDTA treated cultures, indicated a higher amount of Stx produced in the cultures treated with EDTA compared with the non-treated cultures. Assays performed with DH5α(933 W) lysogen showed CD_50_ in dilutions 1/5 when induced with EDTA, while the control (without inducing agent), and cultures treated with mitomycin C did not show cytotoxicity before dilution 1/20. Assays with lysogens C600(933 W) and EDL933 showed similar levels of cytotoxicity with EDTA and mitomycin C and in both cases a CD_50_ one dilution higher than the one observed with the non-induced cultures, in accordance with previous results of mitomycin C assays [8].

Stx1 phages were also induced by EDTA. The influence of EDTA in Stx1 phages induction was also confirmed by PCR (Table 2), by detecting *stx*_1_ in phage DNA extracted from cultures of strain EDL933 induced with EDTA or mitomycin C, but not in the un-induced cultures.

### RecA-independent Stx2 phage induction

DH5α is a strain with a mis-sense mutation consisting of a base pair substitution in the *recA* gene. In addition to the results obtained with DH5α, we constructed an isogenic mutant C600Δ*recA*::*tet*, and after lysogenization with phage 933 W we generated lysogen C600 (933 W)Δ*recA*::*tet*, this mutant was used to confirm the RecA-independent effect of EDTA in 933 W induction. The results confirmed that *recA* is not necessary for EDTA-mediated Stx2 phage induction (Fig. 1D). The number of Stx2 phages in *recA*-mutant and *recA*-mutant complemented with pGEM::*recA* were 5.0×10^5^ and 2.3×10^5^ respectively. Mitomycin C had no effect on phage induction in *recA*-mutant. However, the phage induction with mitomycin C was again observed when the mutation was complemented. Combined use of mitomycin C and EDTA showed similar results to EDTA-mediated phage induction.

### Stx2 phage release from 933 W lysogens

**Figure 2:**
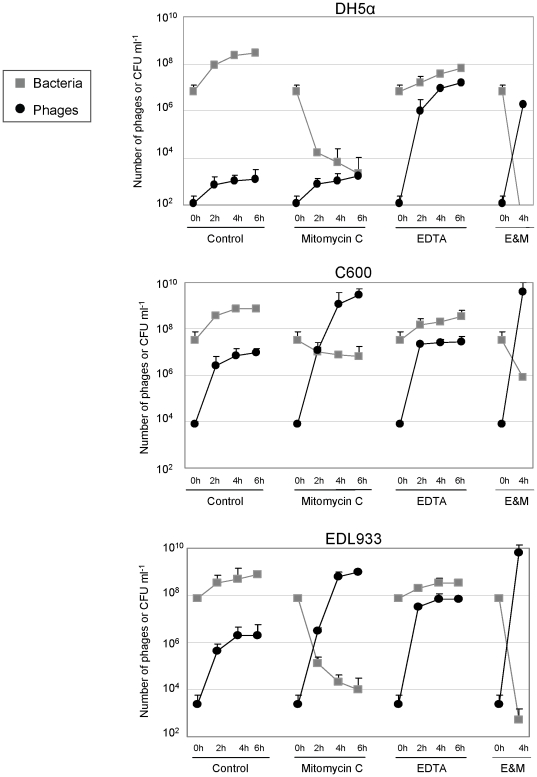
The effect of mitomycin C and EDTA on Stx2 phage induction. Bacteria and 933 W phage evaluation in lysogen cultures incubated in LB broth, LB broth with mitomycin C, LB broth with EDTA and LB broth with EDTA and mitomycin C (E&M) in three strains: (A) DH5α(933 W), (B) C600(933 W) and (C) O157:H7 EDL 933. At the indicated times the numbers of viable bacterial and free Stx phages per ml were determined. Data presented are the mean of three experiments. Error bars indicate the standard deviation.

In this set of experiments we monitored the number of free 933 W phages and viable bacteria in two laboratory lysogens, DH5α(933 W) and C600(933 W), and one EHEC O157:H7 strain EDL933. The results were evaluated and compared after 0, 2, 4 and 6 h of incubation in control cultures and those containing EDTA or mitomycin C (Figure 2). All three bacterial strains showed reduced growth after mitomycin C treatment. In contrast, cultures incubated with EDTA showed an increase in viable cell count over the times tested, although the number of cells detected was lower than in the control cultures for all strains.

The strains C600(933 W) and EDL933 showed a higher level of spontaneous induction, with 1.9×10^6^ and 9.2×10^6^ phages ml^−1^ in 6-h in the respective control cultures. The level of spontaneous 933 W phage induction in *recA*-negative hosts DH5α was low, with a maximum of 1.2×10^3^ phages ml^−1^ in 6-h cultures (Figure 2). As expected, mitomycin C had no effect on 933 W induction in DH5α, though the number of viable cells decreased following treatment with mitomycin C. The number of free 933 W phages detected in the DH5α lysogen culture was significantly higher when this was incubated with EDTA, reaching up to 1.9×10^7^ phages ml^−1^ in 6-h cultures (Figure 2).

The effect of EDTA and mitomycin C on phage induction was visible after 2 h of incubation (Figure 2). EDTA appeared to be a good inductor of Stx phages since all three strains showed an increase in induction and Stx2 phage release when EDTA was used. The number of 933 W phages showed the highest level in 2-h cultures with EDTA, these values were even higher than in cultures with mitomycin C. Comparison of the densities of phages released between cultures incubated with EDTA showed similar induction levels for all strains, ranging from 1.6×10^7^ for DH5α(933 W) to 8.9×10^7^ for EDL933. The addition of mitomycin C increased the number of 933 W phages compared to the control culture over 2, 4 and 6 h in C600(933 W) and EDL933, but not in the *recA*-negative lysogen DH5α(933 W) (Figure 2).

With regards to the three lysogens, C600(933 W) showed the highest number of phages in all conditions tested (control cultures, mitomycin C and EDTA), especially with mitomycin C, where the amount of phages reached a maximum (almost 10^10^ phages ml^−1^). Since there were no significant differences (P>0.05) between the number of Stx2 phages released in 4- and 6-h cultures in any of the conditions tested, 4 h of incubation was used to assess Stx2 phage induction in subsequent experiments.

### The synergistic effect of mitomycin C and EDTA

EDTA affects the membrane structure of bacteria and makes it more permeable and therefore accessible to other antimicrobial agents [38]. We evaluated whether there is a synergistic effect when EDTA and mitomycin C are used in combination. The addition of these two agents together to the three bacterial lysogens significantly reduced (P<0.05) bacterial growth in all strains tested (Figure 2). A total of 7.0×10^7^ phages were released in the culture of EDL933 incubated with EDTA, with 6.1×10^8^ phages released with mitomycin C, and 8.5×10^9^ phages when the two agents were combined. An increase in 933 W phage number was also observed with lysogen C600(933 W). The combination of mitomycin C and EDTA in the cultures of DH5α(933 W) did not increase phage 933 W induction in comparison to the cultures incubated with EDTA only. This was expected, since mitomycin C had no effect on the induction of phage 933 W in this *recA*-negative lysogen. These results are in accordance with those obtained with the isogenic *recA*-mutant (Figure 1).

### Effect of EDTA on Stx2 phage induction in wild-type STEC strains

**Figure 3:**
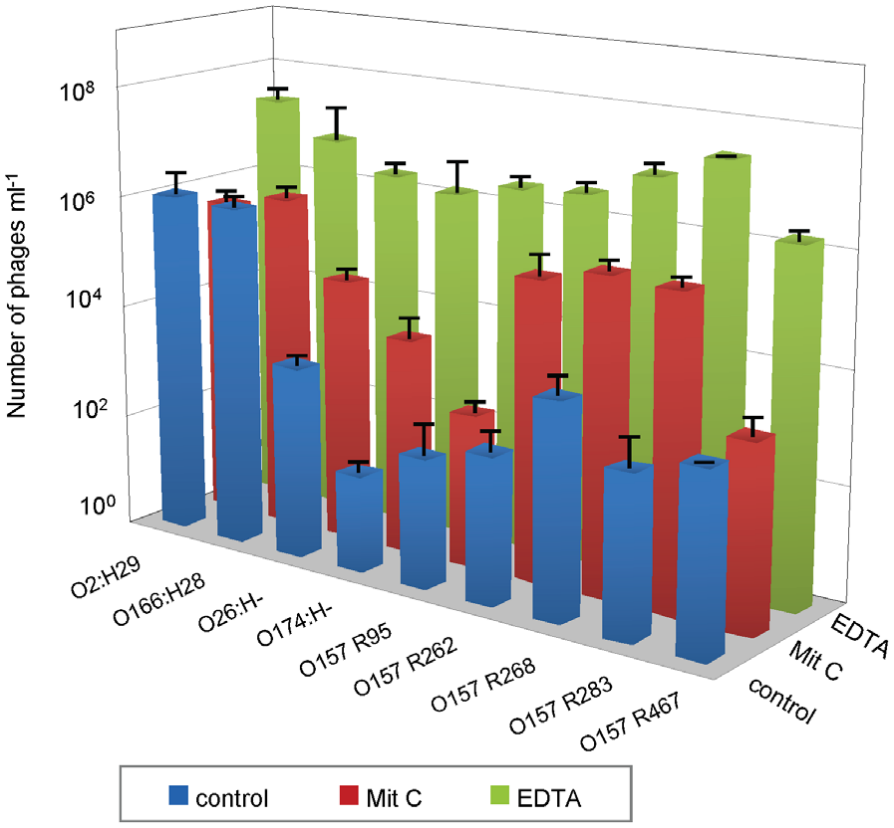
The different inducing capacities of EDTA and mitomycin C in wild-type STEC strains. The differences in Stx2 phage production (ml^−1^) in control cultures and in cultures incubated with mitomycin C or EDTA.

Nine STEC strains positive for the *stx*_2_ gene and negative for *stx*_1_ (Table 1) were used to evaluate Stx2 phage induction with EDTA. These particular strains are clinical isolates that were tested due to the low phage induction observed previously in our lab when using mitomycin C. All 9 strains showed a higher number of phages in cultures incubated with EDTA than in control cultures or those incubated with mitomycin C (Figure 3). Four STEC strains showed no significant differences (P>0.05) in the number of phages present in control cultures and those with mitomycin C. All strains showed a significantly higher level of induction with EDTA than with mitomycin C (P<0.05).

### Effect of RcsA and DsrA on Stx2 phage release

**Figure 4:**
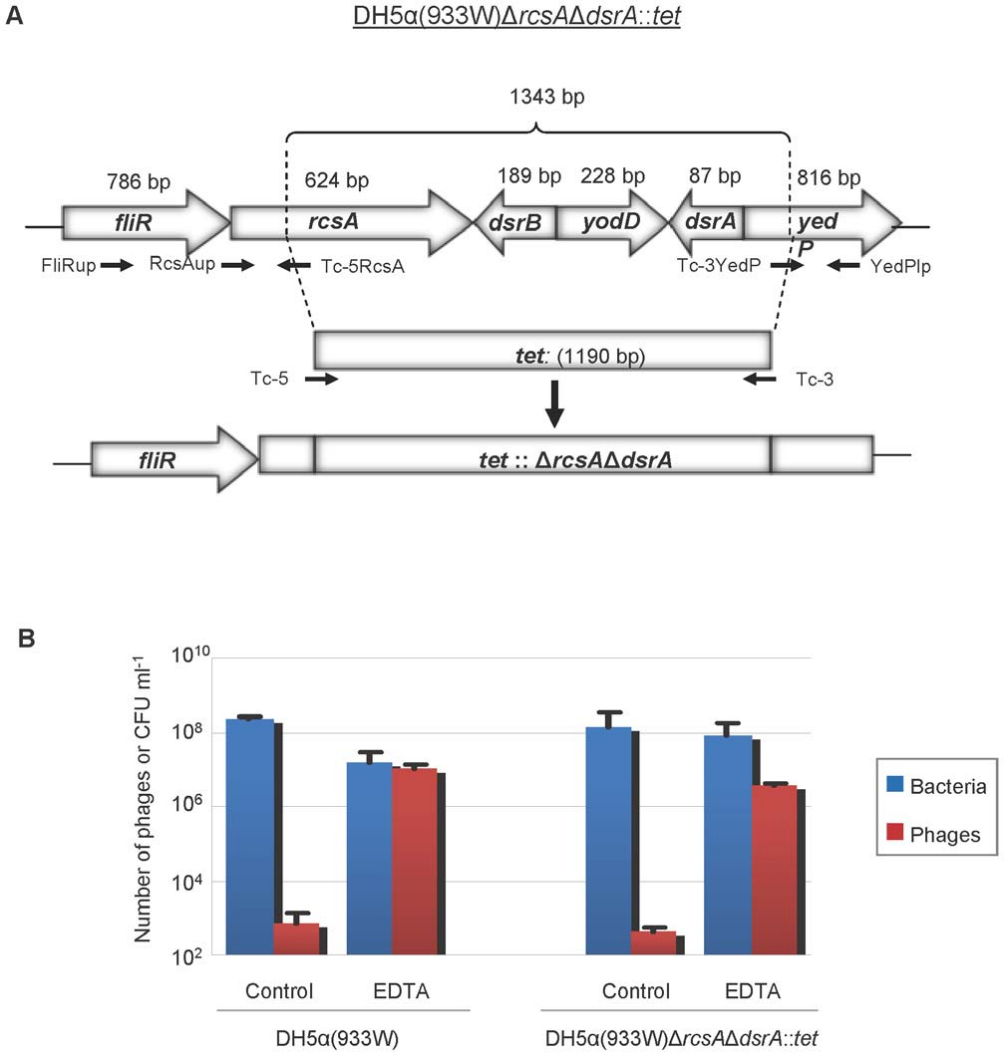
Evaluation of the effect of RcsA and DsrA on Stx2 phage induction. (A) shows the position and direction of the transcription of the *rcsA* and *dsrA* genes and the deletions used to generate mutant DH5α(933 W)Δ*rcsA*Δ*dsrA*::*tet*. The map was drawn based on the distribution in *E. coli* strain K12 MG1655 (GenBank accession n° U00096.2). Their close position on the genome permitted the effect of both genes to be evaluated using a single mutation via the Red Recombinase system. The orientation and positions of primers used to construct mutants with deletions in *rcsA* and *drsA* genes by introducing a *tet* cassette are marked by small arrows. The extent of each deletion is shown but is not drawn to scale. (B) shows the number of 933 W phage and viable bacterial counts of the wild-type strain and the mutant with and without EDTA induction. Data presented are the mean of three experiments. Error bars indicate the standard deviation.

933 W and other described Stx phages belong to the group of lambdoid phages [11]. Therefore, the genes *rcsA* and *dsrA*, which are involved in the induction of λ independently of RecA, were evaluated to determine whether these genes have a role in the induction of 933 W in a *recA*-negative background. For this purpose we constructed the mutant DH5α(933 W)Δ*rcsA*Δ*dsrA*::*tet*, in which both genes were substituted by the *tet* gene, which confers tetracycline resistance (Figure 4A).

The level of spontaneous phage induction or induction in the presence of EDTA was similar (no significant differences, (P>0.05)) in DH5α(933 W) Δ*rcsA*Δ*dsrA*::*tet* and DH5α(933 W) incubated in the same conditions. Thus 933 W phage induction in a *recA*-negative genetic background does not appear to be affected by the lack of *rcsA* or *dsrA* (Figure 4B).

### Effect of envelope stress responses on Stx2 phage induction

**Figure 5:**
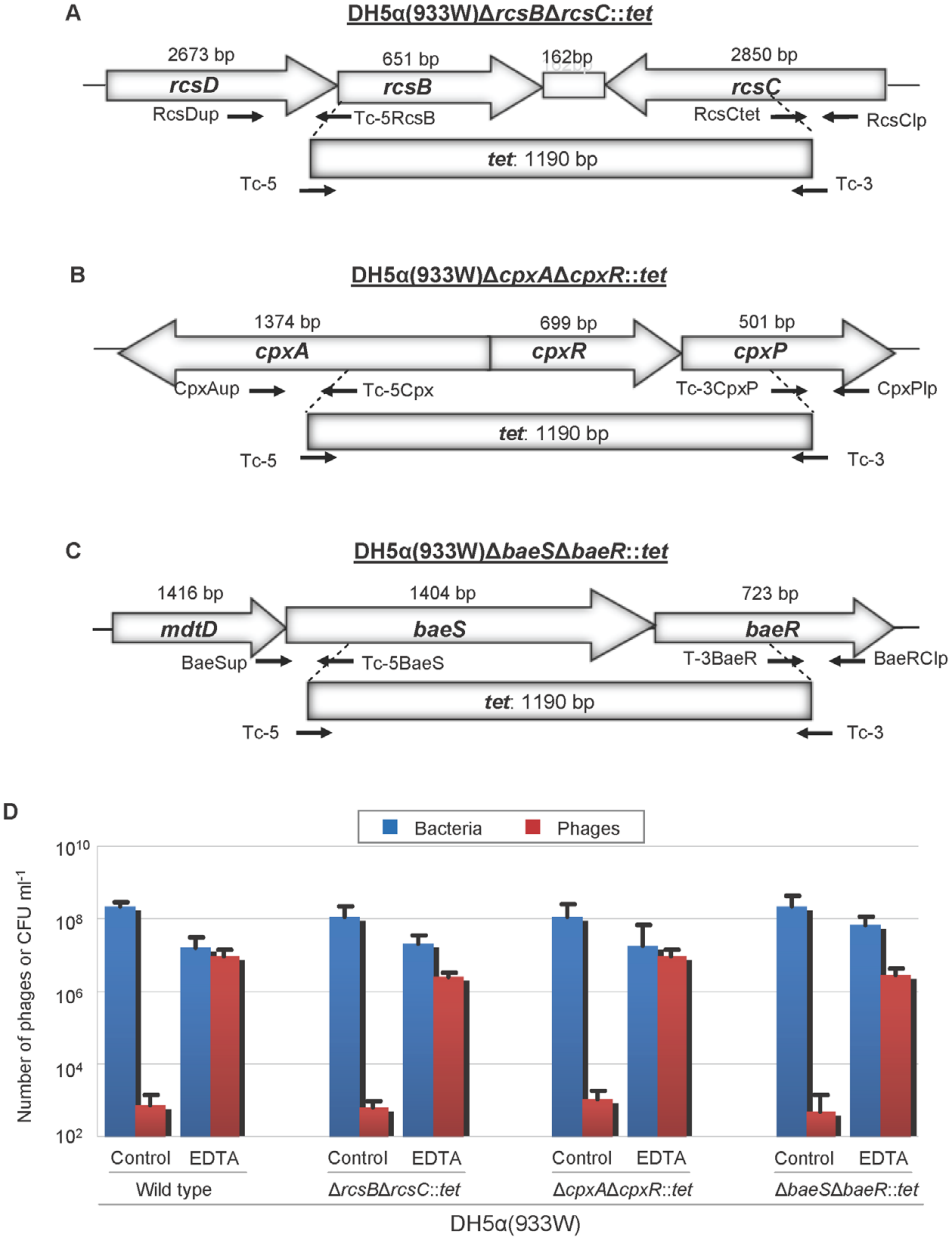
Evaluation of envelope stress pathways in phage induction. The figure shows the position and direction of transcription of the genes composing the (A) RcsBC, (B) CpxAR and (C) BaeSR pathways, and the deletions used to generate mutants DH5α(933 W) Δ*rcsB*Δ*rcsC*::*tet* (A), DH5α(933 W) Δ*cpxA*Δ*cpxR*::*tet* (B), and DH5α(933 W) Δ*baeS*Δ*baeR*::*tet* (C). Schematic representations of the genes mutated for construction of mutants lacking the sensor kinase and response regulator in the selected envelope stress response signalling pathways were drawn based on *E. coli* strain K12 MG1655 (GenBank accession n° U00096.2). The extent of each deletion is shown but is not drawn to scale. The chart (D) shows the number of 933 W phage and viable bacterial counts of the wild-type strain and the three mutants with and without EDTA induction. Data presented are the mean of three experiments. Error bars indicate the standard deviation.

We next evaluated whether the stress exerted by EDTA on the bacterial membrane affected Stx2 phage induction. Bury-Moné and colleagues [39] demonstrated that EDTA triggers three signalling pathways in membrane stress responses: RcsBC, BaeSR and CpxAR. In order to evaluate the involvement of these envelope stress responses in EDTA-mediated Stx2 phage induction, independently of RecA, we constructed the mutants DH5α(933 W) Δ*rcsB*Δ*rcsC*::*tet*, DH5α(933 W) Δ*baeS*Δ*baeR*::*tet*, and DH5α(933 W) Δ*cpxA*Δ*cpxR*::*tet*, disrupting the genes involved in each pathway (Figure 5A–C). Since these are two-component signalling pathways, we inactivated the sensor kinase and response regulator of each pathway by inserting a tetracycline cassette. Lysogenized mutants lacking one of the three pathways showed similar Stx2 phage induction to the wild type DH5α(933 W) in the presence of EDTA (Figure 5D), suggesting that the envelope stress responses were not responsible for RecA-independent 933 W phage induction.

### Effect of chelation on Stx2 phage induction

EDTA is a well-known chelating agent, therefore, our next step was to evaluate whether its chelation effect could cause RecA-independent phage induction. This possibility was evaluated using two approaches: first, by the elimination of cations that could reproduce the EDTA chelating effect from the media; second, by adding other chelating agents. Stx2 phage induction mediated by EDTA was not due to the lack of cations in the medium. When the DH5α(933 W) lysogen was grown in AB minimal medium, each time lacking one of the cations, Mg^2+^ or Ca^2+^ or Fe^3+^, there were no significant differences (P>0.05) in the number of phages induced (Table 3).

**Table 3:** Effect of chelation of 933 W induction.

Condition	Growth medium	Incubation time (h)	933 W phages ml^−1^	SD	Phage increase (log_10_)*
*Control*	*LB*	*0*	*1.2×10^2^*	*1.1×10^2^*	*-*
Control (spontaneous induction)	LB	4	1.1×10^3^	6.8×10^2^	0.96
EDTA	LB	4	9.7×10^6^	2.0×10^6^	4.91
Sodium citrate	LB	4	4.1×10^6^	4.7×10^6^	4.53
2′2 dipyridyl	LB	4	2.6×10^2^	2.5×10^2^	0.34
TPEN	LB	4	2.0×10^2^	1.5×10^2^	0.22
BAPTA	LB	4	2.3×10^4^	3.9×10^4^	2.28
EGTA	LB	4	4.1×10^3^	4.5×10^3^	1.53
EDTA+Ca^2+^	LB	4	3.3×10^2^	3.4×10^2^	0.44
EDTA+Mg^2+^	LB	4	2.1×10^2^	2.0×10^2^	0.24
EDTA+Fe^3+^	LB	4	1.9×10^1^	2.0×10^1^	−0.80
*Control*	*AB*	*0*	*1.0×10^2^*	*9.3×10^1^*	*-*
Control (spontaneous induction)	AB	16	2.3×10^3^	2.6×10^3^	1.36
EDTA	AB	16	5.6×10^5^	6.0×10^5^	3.75
Sodium citrate	AB	16	8.4×10^4^	8.3×10^4^	2.92
Δ Ca^2+^	AB	16	1.9×10^2^	2.2×10^2^	0.28
Δ Mg^2+^	AB	16	8.7×10^1^	9.2×10^1^	−1.42
Δ Fe^3+^	AB	16	2.0×10^2^	1.8×10^2^	−1.06

• Log_10_ variation in the number of phages related to the control strain without induction at time 0.

The effect of Ca^2+^, Mg^2+^ and Fe^3+^ cations as well as chelating agents (EDTA, sodium citrate, 2′2 dipyridyl, TPEN, BAPTA and EGTA) in 933 W phage induction from lysogen DH5α(933 W). Induction was evaluated in minimal AB medium and in LB broth. Control cultures without inductors at time 0 are shown in italics.

The next set of experiments yielded more informative results. We used five other chelating agents: 2′2 dipyridyl, which is an Fe^2+^ chelator; sodium citrate which, like EDTA, is a chelator of Ca^2+^ and Mg^2+^; BAPTA and EGTA, chelators with a higher affinity for Ca^2+^ than for Mg^2+^; and TPEN, a Zn^2+^ specific chelator. 2′2 dipyridyl, EGTA and TPEN did not induce a differential response in phage induction, while BAPTA produced a slight increase, which was lower than that caused by EDTA or sodium citrate. These results suggest that that chelation of Fe^2+^, Ca^2+^ or Zn^2+^ is not the main factor responsible for phage induction. Results observed with sodium citrate, which had the same effect as EDTA, and to a lesser extent, BAPTA, indicated that chelating properties were responsible for RecA-independent Stx phage induction and that Mg^2+^ is a plausible candidate.

However, the question now arose as to why the lack of cations did not cause the same effect as the presence of EDTA. One possible explanation could be the chelating effect of EDTA (or sodium citrate) on the cations present in the bacterial membrane, which is independent of the cations available in the medium. To test this, we saturated the EDTA chelating properties by adding Ca^2+^, Mg^2+^ or Fe^3+^ to the medium. In this case, the effect of EDTA on phage induction was abolished (Table 3), thus confirming our hypothesis. Similar inhibition of induction was observed when using sodium citrate in a medium supplemented with the different ions (data not shown).

### pH affects Stx2 phage induction

**Figure 6:**
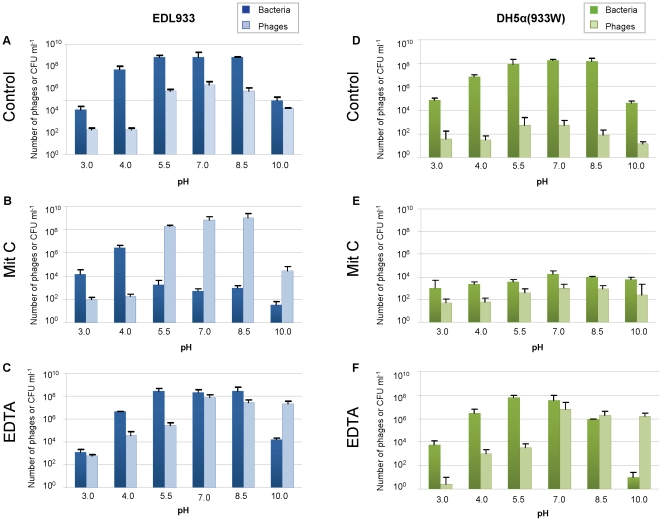
Effect of pH on Stx2 phage induction. Number of Stx2 phage and viable bacterial counts in EDL933 (A, B and C) and DH5α (933 W) (D, E and F). Cultures were evaluated without inducing agent (A and D), and with inducing agents: mitomycin C (B and E) and EDTA (C and F). The number of phage and viable bacterial counts in cultures were determined after incubation at 37°C for 4 h. Data presented are the mean of three experiments. Error bars indicate the standard deviation.

During this study, we observed some discrepancies in phage induction due to pH levels. For this reason the pH of EDTA was always adjusted to 7.0 in the previous experiments. Some influence of pH on phage induction was suspected and this was assayed in the following set of experiments. We used cultures of DH5α(933 W) and EDL933 over a broad pH range (3.0, 4.0, 5.5, 7.0, 8.5 and 10). The number of free 933 W phages and viable cell counts were evaluated in control cultures and after induction with mitomycin C or EDTA (Figure 6).

Our first observation was that the host strains survived with EDTA similarly to the control without inducing agent, whereas bacterial cells showed lower survival with mitomycin C at pH ranging from 5.5 to 10 (Figure 6). At pH 3 and pH 4, similar bacterial survival was observed for both conditions in cultures of EDL933 and DH5α(933 W). Low pH (3.0 and 4.0) affected bacteriophage induction, since phages were poorly induced from all cultures, regardless of the inducing agent or the strain (Figure 6). As pH rose, the number of phages increased, to a maximum at pH 7. At pH 10 the numbers of phages decreased again in both strains, more dramatically in control cultures and those treated with mitomycin C. This effect was reduced in cultures treated with EDTA at pH 10. At pH from 5.5 to 8.5, the level of spontaneous phage induction (controls) as well as the number of phages induced with mitomycin C or EDTA was higher than at more extreme pH values. The addition of mitomycin C had no effect on phage induction at any point in the pH range tested in the *recA*-negative strain DH5α (933 W).

At pH 5.5–8.0, more phages were detected with mitomycin C than with EDTA in EDL933. However, at more extreme pHs the induction level was higher with EDTA than with mitomycin C in EDL933. Comparison was not possible with DH5α. For DH5α, an acidic environment caused a decrease in the number of phages induced with EDTA, while in neutral and alkaline conditions (pH 7, 8.5 and 10), the number of phages induced was higher than in the control and with mitomycin C, and showed no significant differences compared with EDL933 (P>0.05).

## Discussion

Phage induction is a complex process dependent on bacterial and phage factors [46], [47]. Strains lysogenized with Stx phages represent a different genetic background and this is reflected in their phage-inducing capacity even though they carry the same phage in their genome [9], [47]. The strains involved more frequently in outbreaks and severe diseases in humans are found to be induced more easily and to produce higher amounts of Stx and Stx phages [47], [48]. Surprisingly, in this study, the highest amount of Stx2 phages was detected when the laboratory strain C600(933 W) was used as a host. The fact that 933 W phage number was higher in a laboratory lysogen than in the original EHEC host strain EDL933 could be a consequence of the presence of other lamboid phages in the genome of EDL933 [49]. As observed previously, strains lysogenized with several phages showed lower levels of Stx phage induction [50], [51].

In addition to the differences in inducing capacity between strains, Stx2 phage induction with mitomycin C is not an intrinsic feature of all O157 STEC. Other authors reported that some STEC strains failed to produce Stx phages under mitomycin C treatment even though they appeared to be carrying functional phages [52]. Since the replication of the phage genome and phage cell lysis are involved in Stx production and release outside the cell, it remains unclear whether in these cases *stx* genes play an active role in pathogenesis. Here we tested STEC strains from our collection that do not produce Stx2 phages, or that produce them in a very low number after exposure to mitomycin C. Real-time PCR showed that most of the strains induce phage spontaneously at a very low level, while mitomycin C failed to induce Stx2 phages. In this study, the level of spontaneous 933 W phage induction was dependent on the strain used, and, as expected was high in *recA*-positive strains. In contrast to the variability in the inducing capacities of mitomycin C in this study and others [52], all strains tested showed an increase in the number of Stx2 phages when incubated with EDTA. The differences in Stx2 phage induction with EDTA and mitomycin C may be attributed to activation of different pathways that lead to activation of the phage lytic cycle.

EDTA affects the bacterial membrane, which it renders more permeable to other antimicrobial agents [38]. The use of EDTA to inhibit EHEC O157:H7 in combination with other agents has been evaluated by several authors [36], [37]. However, the potential inducing properties of the antimicrobial agent are not usually considered. If the agent applied induces Stx2 prophages, it can increase the amount of Stx produced and the number of free phages in a given environment even when the number of bacteria decreases. In turn, this can increase the probability of transmitting the *stx* gene to other susceptible bacteria.

Although the influence of several factors on *stx*_2_ expression has been studied [18], [22], [23], [26], [27] the induction of Stx2 phages independently of RecA has not been described. RecA-stimulated phage repressor cleavage was reported to be necessary for Stx phage induction (including spontaneous phage induction) after exposure to inducing agents such as mitomycin C [20], [28]. The possibility of an alternative pathway of Stx2 induction that is independent of RecA was considered by our group when a lysogen of the *recA*-negative strain DH5α was successfully used as a donor of Stx2 phage. The phage induction in a *recA*-negative background may be a consequence of repressor perturbation, which could activate the lytic pathway [19], [53].

The finding that EDTA induced phages from *recA*-negative strains, verified with DH5α as well as with an isogenic mutant, led us to explore the possible mechanisms underlying this induction. Since Stx phages belong to a group of lambdoid phages, the molecular mechanism behind RecA-independent induction of λ was evaluated first. A positive transcriptional regulator of exopolysaccharide synthesis, RcsA, was shown to be directly involved in RecA-independent induction of λ [30], [32]. In addition to RcsA, DsrA also seems to contribute to the induction of λ [30]. Our results indicate that there are differences in induction patterns between λ and Stx2 phage 933 W, since EDTA-mediated 933 W phage induction did not appear to be mediated by either factor. However, due to the considerable genetic heterogeneity among Stx phages [11], we cannot rule out the possibility that the induction of some other Stx phages might follow patterns that are more similar to λ.

For *E. coli*, six known envelope stresses are the first in a chain of environmental signals that are responsible for monitoring and maintaining cell envelope integrity [54]. In theory at least, it is possible that, for certain stress responses, a set of genes are activated in such a way that, if host cell survival is threatened, the prophage is induced. On that basis, several possible molecular mechanisms that might affect EDTA-mediated phage induction were evaluated, since the EDTA affects the bacterial outer membrane and causes stress [38]. Buri-Moné et al. [39] reported that three envelope response pathways are induced after *E. coli* exposure to EDTA: RcsBC, CpxAR and BaeSR. The RcsBC and CpxAR pathways have been also implicated in expression virulence factors in *E. coli* [54]. However, our results indicate that none of these three pathways is responsible for EDTA-mediated Stx phage induction.

EDTA is widely used as a chelating agent as it can form complexes with a wide range of metals [34]. We concluded that chelation and the increase in Stx2 phage induction are linked, since sodium citrate, another chelating agent, showed the same effect on Stx2 phage induction as EDTA in a RecA-negative background. As expected, the chelation of iron had no effect on induction, which is consistent with other authors [23]. However, Ca^2+^ and Mg^2+^ play a crucial role in stabilizing the structure of the outer bacterial membrane [38]. One way in which EDTA acts on the bacterial cell is by disrupting the structure of the membrane by complexing the Ca^2+^ and Mg^2+^ cations present in the membrane [38]. Thus, capture of these cations and the consequent damage to the outer membrane caused by EDTA could lead to Stx2 phage induction. Interestingly, the effect of EDTA was inhibited in a medium supplemented with cations, although the lack of cations in the same medium did not result in phage induction. It is possible that EDTA is blocked by the binding of cations (Ca^2+^, Mg^2+^ and Fe^3+^), preventing its chelating effect on bacterial cells and subsequent phage induction. This study did not examine whether the application of these cations reduces Stx2 phage induction *in vivo*, but interestingly, a recent report shows that Stx toxin expression is inhibited by zinc *in vivo* in the intestinal loop in rabbits [55].

Furthermore, EDTA might affect DNA binding by the phage repressor, but this would mean that EDTA enters the bacterial cell, and it is not clear that this is the case. The effect of osmotic pressure and changes in the concentration of intracellular ions in phage induction should also be considered. Previous reports showed that a Na^+^ increase in the medium increased the intracellular Na concentrations in the bacteria. This increase affects λ^imm434^ induction in a way that is independent of RecA, RcsA and DsrA, and could be due to variations on the repressor's affinity for DNA [31]. However, it has been demonstrated that the increase in Na^+^ concentration does not affect Stx2 phage induction [56]. Other studies [26] showed that under starvation conditions, *E. coli* cells undergo global modification of their protein expression, which might increase resistance to chelating agents. Our observations are partially in line with reports that starvation impaired prophage induction and lysis in lambdoid phages [26].

Another determining factor for Stx2 phage induction is pH. The inhibition of Stx production reported by probiotic bifidobacteria was attributed in part to low pH [57]. We have demonstrated a role of pH by inhibiting Stx phage induction at low pH. Addition of mitomycin C at pH<5.5 preserved bacteria from phage lysis and the survival of the lysogen was higher in acidic environments. This reduction of phage lysis at low pH is an important factor in bacterial virulence, since resistance to acidity of the stomach, an important characteristic of STEC, detemines their capacity to colonize the human gut [2], [4]. Little [58] reported that λ phage repressor undergoes self-cleavage at high pH and that this process is accelerated in the presence of EDTA. High pH favoured Stx2 phage induction with EDTA in a *recA*-negative background and the effect was observed even at pH 10. The fact that neutral and alkaline conditions favoured phage induction might explain the release and action of Stx in the colon, where pH ranges from 7 to 8.

Both the dissemination of *stx* genes and regulation of their expression are matters of interest in public health. EDTA is a major organic pollutant in the environment because of its widespread usage in a variety of industrial processes and its resistance to biodegradation [59]. In addition, the frequent use of EDTA and citrate in food and pharmaceutical products is of concern since this might increase the risk of Stx production if ingested, and could contribute to the increased load of Stx phage in food. Ingested Stx phages or those in food stuff could then convert other susceptible *E. coli* strains and contribute to a higher risk of STEC infections. Inducing agents and conditions that activate the lytic cycle of the Stx phage could affect the outcome and severity of infection with STEC and could contribute to increased phage-mediated horizontal *stx* gene transfer.

## Supporting Information

Figure S1 **A model of possible effects of EDTA on Stx2 phage induction.** RcsA is an unstable positive regulator of colanic acid synthesis in *E. coli*. The overexpression of *dsrA*, a small RNA, prevents the degradation of RcsA. Both RcsA and DsrA have been implicated in Rec-independent induction of phage λ (A). RcsA-mediated induction of λ required RcsB. RcsB is part of the phosphorelay system of the membrane stress response system (RcsBC). RcsBC responds to a number of environmental stimuli and has a role in biofilm formation (EDTA affects the bacterial biofilm). In addition to RcsBC, *E. coli* possesses several other envelope stress response systems that detect changes in the environment and redirect gene expression. Three of these envelope stress responses are activated by EDTA: RcsBC, CpxAR and BaeSR (B). The addition of EDTA to the bacterial culture can result in the chelation of cations from the growth medium (Ca^2+^, Mg^2+^ or Fe^3+^) or the chelation of divalent cations (Ca^2+^ and Mg^2+^) from the bacterial cell envelope (C). The effect of EDTA as a chelating agent is strongly dependent on pH (D).
(TIF)
