# Correction: Characterizing RecA-Independent Induction of Shiga toxin2-Encoding Phages by EDTA Treatment

**DOI:** 10.1371/journal.pone.0193475

**Published:** 2018-02-21

**Authors:** Lejla Imamovic, Maite Muniesa

In Table 2, the sequence of oligonucleotide STX-Any r is incorrect. Please see the corrected Table 2 here.

**Table 2:** Oligonucleotides used in this study.

Name	Sequence (5’- 3’)	Characteristics	Reference
Stx1-UP Stx1-LP	CAGTTAATGTGGTGGCGAAGG ACTGCTAATAGTTCTGCGCATC	gene *stx*_1_-A+B	[16]
S2Aup S2Alp	ATGAAGTGTATATTATTTA TTCTTCATGCTTAACTCCT	*stx*_2_*A* fragment	[9]
GK3 GK4	TCAGCCCCATACGATATAAG TGGAGTGGTGAATCCGTTAG	*stx*_2_*B* fragment	[16]
UP378 LP378	GCGTTTTGACCATCTTCGT ACAGGAGCAGTTTCAGACAG	*stx*_2_*A* fragment	[42]
Tc-5 Tc-3	TCAGCCCCATACGATATAAG TGGAGTGGTGAATCCGTTAG	gene *tet* (Tc)	[44]
Tc-int	TGTCGGAATGGACGATAT	binding 5’of *tet* cassette	[44]
RR46 LP RR46-UP	GAGCTCTAAGGAGGTTAT GTGCAGTACTCATTCGTT	gene Red recombinase in vector pKD46	[44]
pBADf pBADr	ATGCCATAGCATTTTTATCC GATTTAATCTGTATCAGG	Confirmation of pBAD construct	Invitrogen
pGEM7up	TGTAATACGACTCACTAT	Confirmation of pGEM construct	Promega
RecX up RecAv lp	TCAGTCGGCAAAATTTCGC AGGTGCACTGAAAGCGGCTCG	*recA* gene	This study
Tc-5RecX lp Tc-3RecAvup	CTAACGGATTCACCACTCCACGCAGTAGCCTGACGACGCA CTTATATCGTATGGGGCTGAGGACCTGCGCGTATTCGCCA	fragment of the *tet* gene for generation of Δ*recA* mutant	This study
Tc-5RcsB Tc-3RcsC	CTTATATCGTATGGGGCTGAGCGAATACCGAACAAGACTA CTAACGGATTCACCACTCCATCACTACCGCTCTGCCCTGC	fragment of the *tet* gene for Δ*rcsB*Δ*rcsC* mutant	This study
RcsAup YedPlp	ATGTCAACGATTATTATGGAT TGGCTAATACCTGAGATGAT	fragment between *rscA* and *dsrA* genes	This study
Tc-5RcsA Tc-3YedP	CTTATATCGTATGGGGCTGACGCTGACTGTTAGAAGCATC CTAACGGATTCACCACTCCAATGTTTTCAATTCAACAACC	fragment of the *tet* gene for Δ*rcsA*Δ*dsrA* mutant	This study
CpxAup CpxPlp	GAATAACGCAGAGCATTAC GAACTGACTGCCAGCGTTGA	CpxAR phosphorelay system	This study
Tc-5CpxA Tc-3CpxP	CTTATATCGTATGGGGCTGAAGTTGTGGAGTGAAGTGCTG CTAACGGATTCACCACTCCATGAAGCCTTCCATCTCGAG	fragment of the *tet* gene for Δ*cpxA*Δ*cpR* mutant	This study
BaeSup BaeRlp	TGAAGTTCTGGCGACCCGGTAT CTAAACGATGCGGCAGGCGTCG	BaeSR phosphorelay system	This study
Tc-5BaeS Tc-3BaeR	CTTATATCGTATGGGGCTGACTGAAAGACAAAGCGATCAT TAACGGATTCACCACTCCAGCGTAGTAACCGACCGCACC	fragment of the *tet* gene for Δ*baeS*Δ*baeR* mutant	This study
STX-Any f STX-Any r STX-Any M	ACGGACAGCAGTTATACCACTCT CTGATTTGCATTCCGGAACGT FAM- CCAGCGCTGCGACACG-NFQ	Real-Time PCR for *stx*_2_ gene detection	[45]
