# Supplementary figures and images for: Characterizing RecA-Independent Induction of Shiga toxin2-Encoding Phages by EDTA Treatment
Source: PLoS One. 2012 Feb 29;7(2):e32393. doi: 10.1371/journal.pone.0032393 (PMC3290563; doi:10.1371/journal.pone.0032393)

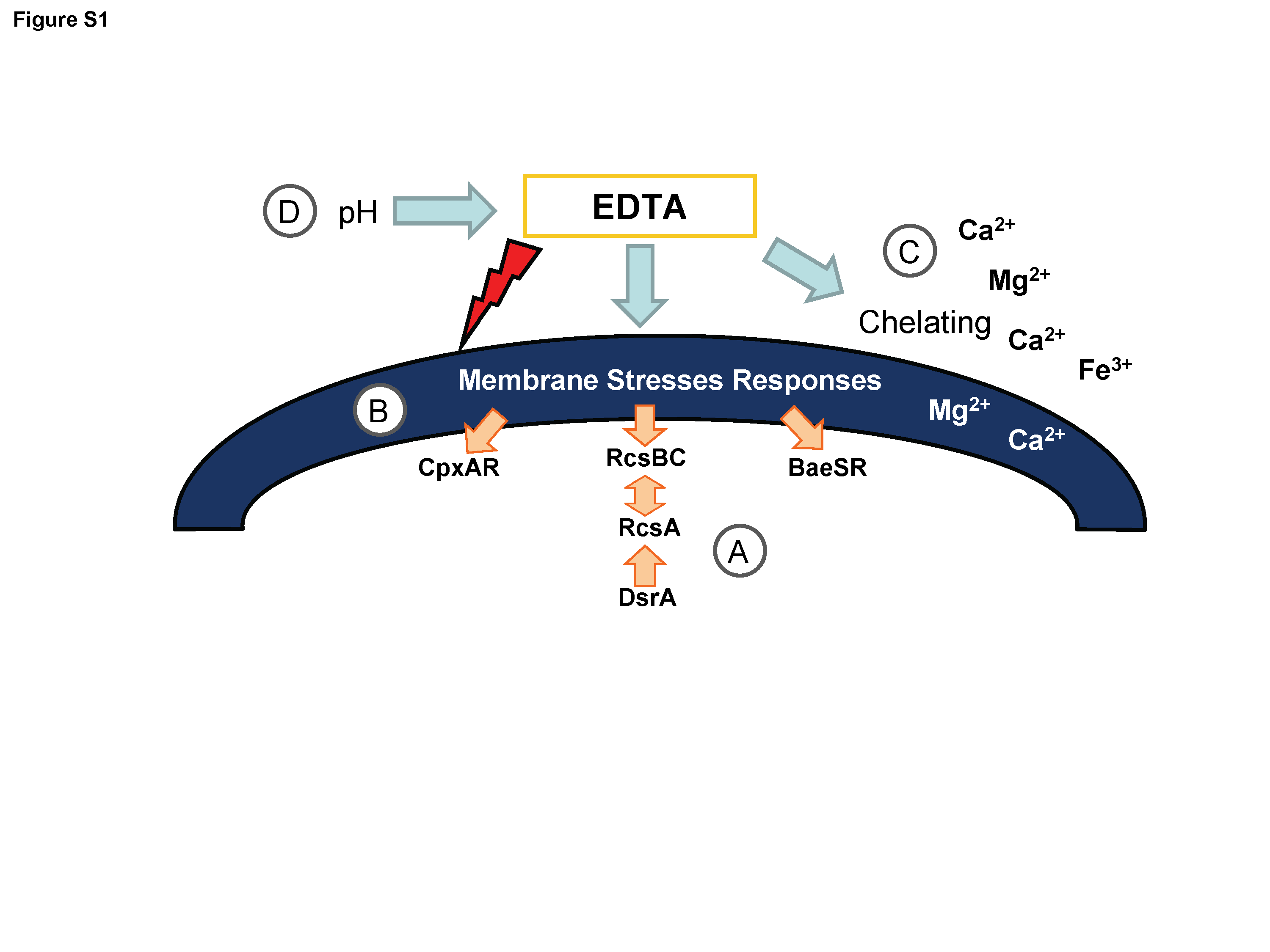

Supplement: Figure S1 — A model of possible effects of EDTA on Stx2 phage induction. RcsA is an unstable positive regulator of colanic acid synthesis in E. coli. The overexpression of dsrA, a small RNA, prevents the degradation of RcsA. Both RcsA and DsrA have been implicated in Rec-independent induction of phage λ (A). RcsA-mediated induction of λ required RcsB. RcsB is part of the phosphorelay system of the membrane stress response system (RcsBC). RcsBC responds to a number of environmental stimuli and has a role in biofilm formation (EDTA affects the bacterial biofilm). In addition to RcsBC, E. coli possesses several other envelope stress response systems that detect changes in the environment and redirect gene expression. Three of these envelope stress responses are activated by EDTA: RcsBC, CpxAR and BaeSR (B). The addition of EDTA to the bacterial culture can result in the chelation of cations from the growth medium (Ca2+, Mg2+ or Fe3+) or the chelation of divalent cations (Ca2+ and Mg2+) from the bacterial cell envelope (C). The effect of EDTA as a chelating agent is strongly dependent on pH (D). (TIF) [file pone.0032393.s001.tif]
